# Medical Guidelines and Performance Measures: The Need to Keep Them Free of Industry Influence

**DOI:** 10.4103/0973-1229.33005

**Published:** 2008

**Authors:** Peter Q. Eichacker, Charles Natanson

**Affiliations:** * *Critical Care Medicine Department, National Institutes of Health, Bethesda, MD*; ** *Clinical Center, National Institutes of Health, Bethesda, MD*

To be unbiased and represent best practice, medical guidelines should be free of industry influence, carefully vetted among experts in the field and encompass treatments widely accepted based on difficult to refute evidence (Shaneyfelt, Mayo-Smith and Rothwang, 2006; Grilli, *et al.*, 2000; Grissom, 2000). These requirements are even more important if individual guideline recommendations are to be grouped into “bundles” to serve as performance measures and provide a possible basis for reimbursement. In a fall 2006 issue of the *New England Journal of Medicine*, we presented our concerns when these requirements were not met (Eichacker, Natanson and Danner, 2006). As an example, which directly affected us as physicians in the field of critical care medicine, we described the close relationship between the development and implementation of the Surviving Sepsis Campaign guidelines and the marketing efforts of Eli Lilly and Company, the primary financial sponsor for this campaign. Considering solutions to such a problem in the context of our original concerns is worthwhile.

## RhAPC: Evidence *vs* Marketing

Recombinant human activated protein C (rhAPC) is a drug manufactured by Eli Lilly for the treatment of severe sepsis, a condition commonly cared for in the intensive care unit (Bernard *et al.,* 2001). Although rhAPC was reported to significantly improve survival in the original phase 3 trial testing it [the Recombinant Activated Human Protein C Worldwide Evaluation in Severe Sepsis (PROWESS) trial], questions arose about the trial and the risk-benefit ratio of the agent itself during evaluation by both the United States Food and Drug Administration (FDA) and the European Medicines Agency (EMEA) (Bernard *et al.*, 2001; Warren *et al.*, 2002; Siegel, 2002). Half of the FDAs advisory panel voted that a confirmatory trial be performed prior to approval. Ultimately however the two agencies did approve rhAPC but restricted it to patients with a high risk of death and requested further trials to define the agent's effects in specific subgroups (BioPortfolio, 2007).

Persistent questions surrounding rhAPC resulted in initial sales far less than had been predicted (Regaldo, 2003; National Institutes of Health, 2007). Eli Lilly then embarked on a marketing effort for which it hired Belsito and Company, a public relations firm, to improve sales (Slade, Tamber and Vincent, 2003). There were several components to this programme, including one to establish a Surviving Sepsis Campaign to raise awareness of severe sepsis and generate momentum toward the development of treatment guidelines. After announcement of the campaign at the European Society of Intensive Care Medicine's (ESICM) annual meeting in 2002, the second phase to develop guidelines for the treatment of sepsis was initiated in June 2003 (Dellinger, *et al.*, 2004; Landucci, 2004). A committee of experts met and, within several months, a set of guidelines was formulated and then published in *Critical Care Medicine* in March 2004 (Landucci, 2004). Lilly provided more than 90% of the funding for these two phases and a substantial number of participants had relationships with the company. Employing an evidence-based grading system which did not account for the level of controversy surrounding particular therapies, rhAPC was given one of the highest ratings (Fourrier, 2004). In addition, a supplement published eight months after the guidelines themselves made no mention of newly available clinical trial data further questioning rhAPCs efficacy but confirming its risk of serious bleeding (Levy, *et al.,* 2004).

Following guideline publication, a third phase of the Surviving Sepsis Campaign was initiated which incorporated selected recommendations into two treatment bundles to serve as performance measures (Townsend *et al.*, 2005; Eichacker *et al.,* 2005). Despite increasing published opinion that rhAPC undergo additional testing to clearly define the patient population it might be effective in, this treatment was included in one bundle (Mackenzie, 2005; Wiederman, 2005; Friedrich, 2006; LaRosa, 2006; Baillie, 2006; Society of Critical Care Medicine, 2007). Once again, this phase was supported by unrestricted educational grants from Eli Lilly while many of its participants had reported financial or other relationships with the company (Eichacker *et al.,* 2005). In addition Lilly provided funding for workshops, both nationally and internationally and the publication of a quarterly journal, which promoted this phase of the campaign (McBride, 2006; Report on the Surviving Sepsis Campaign Roadshow, 2007; Pronovost and Berenholtz, 2007). Very concerning, the campaign sought to collaborate with public, non-profit organizations such as the Voluntary Hospital Association, the Institute for Healthcare Improvement and the Joint Commission on Accreditation of Healthcare Organizations in the implementation of these bundled recommendations (Studdert, Mello and Brennan, 2004; Osborn, 2006; IHI, 2007).

## The Dilemma

Informative clinical guidelines and their implementation as performance measures may well benefit patients. Many who contributed to the Surviving Sepsis Campaign surely believed that it was a way to reduce sepsis-related deaths. However, examined in the context of marketing efforts by Eli Lilly, this guideline process appears to have been overtaken for commercial purposes. We concluded in our Perspectives that:

Professional societies and other stakeholders must work together to promote a consistent guideline-development process, a robust rating system for guidelines that is applicable to all subspecialties and a policy that prohibits the pharmaceutical and medical-device industries from directly or indirectly funding or influencing practice standards. The challenges involved in producing first-rate guidelines and performance standards are only exacerbated by the intrusion of marketing strategies masquerading as evidence-based medicine (Eichacker, Natanson and Danner, 2006).

The problems outlined here are not unique to critical care medicine (Kassirer, 2004; Schunemann, Fretheim and Oxman, 2006; Krumholz *et al.*, 2007; Harris and Roberts, 2007). While collaboration between the medical profession and industry can be a productive process and one that is essential for the development and delivery of new therapies, ensuring that the ultimate goals of each group do not conflict is paramount.

## Remedies

At this time there are several ways in which efforts by the Surviving Sepsis Campaign could be strengthened. First, a guideline process should be instituted that accounts not only for the type of evidence supporting an agent or medical device but also the debate and questions that pertain to that evidence (Fourrier, 2004). Furthermore, data should be provided regarding the level of consensus reached among guideline committee members on specific recommendations. Measures such as these are recognized to be critical elements in the formulation of high quality guidelines (Oxman, Schunemann and Fretheim, 2006; Masur, 2007). Second, funding for guideline formulation should be done free of industry support (Brennan *et al.*, 2006; Steinbrook, 2007). Direct professional society support for the Surviving Sepsis Campaign would begin to solve this problem (Steinbrook, 2007). However, companies that originally provided financial support for the Surviving Sepsis Campaign still provide these professional societies with unrestricted educational grants for symposia and other educational forums. Measures analogous to those recommended by Brennan *et al.* (2006) for academic medical centres could help if these societies are to interact with industry while also providing financial support for the Surviving Sepsis Campaign (Taylor and Giles, 2005). This would include rules preventing the use of any one company's funding for specific activities and a central repository for industrial funding that would allow later distribution of resources free of the influence of any one donor. Most importantly however “… the amount of funds contributed (by individual manufacturers) and the eventual use of such funds should be posted (regularly) on a publicly available web site,” (Taylor and Giles, 2005). Finally, mechanisms such as those in used by the American Heart Association should be in place to ensure that physicians with relationships to companies standing to profit from guideline recommendations not serve as chairman or voting members of the guideline committee (Elliott, 2004; Methodology Manual for the ACC/AHA Guideline Writing Committees, 2007). Such a policy should also apply to programmes designed to implement industry supported guidelines as quality indicators or performance measures. Disclosure alone is likely to be insufficient to allay concerns regarding the influence such relationships might have on the guideline process (Bero, Glantz and Hong, 2005).

Guidelines are an established part of medicine today. Their use not only in the care of individual patients but also, correctly or not, as measures of performance, is likely to grow. Ensuring that guidelines provide an objective appraisal of all available data, free of possible influence by industry, is essential for serving the best interests of patients and healthcare workers alike.

## About the Authors

Peter Q. Eichacker is currently Senior Investigator and Head of the Critical Care Medicine Section in the Clinical Center, NIH, Bethesda, MD. He earned an undergraduate degree from Boston University and then a medical degree from New York University. He completed a residency and chief residency in Internal Medicine followed by a fellowship in Pulmonary Medicine at Albert Einstein Hospital and College of Medicine in New York. In 1986

Dr. Eichacker joined the National Institutes of Health in Bethesda, MD as a fellow in the Critical Care Medicine Department. He was then appointed as a Senior Investigator and Head of the Critical Care Medicine Section at the Clinical Centre at the NIH in 1991. His primary areas of research have been in the pathogenesis and treatment of septic shock and sepsis induced lung injury related to commonly encountered types of bacteria as well as to B. anthracis.

Charles Natanson is currently Senior Investigator and Head of the Anesthesiology Section in the Clinical Centre, NIH, Bethesda, MD. He earned an undergraduate degree from the University of Wisconsin-Madison and then a medical degree from Columbia University of Physicians and Surgeons. He completed a residency in internal medicine at New York Hospital Cornell Medical Center followed by one in anaesthesiology at the University of California, San Francisco. In 1981 Dr. Natanson joined the National Institutes of Health in Bethesda, MD as a senior investigator with the Critical Care Medicine Department in the Clinical Centre. His primary research interests include using large and small animal models of sepsis to study the pathogenesis of and new therapeutic strategies for, septic shock.
